# Leveraging 3D Heart Visualisation and Data Balancing Techniques for ECG Classification

**DOI:** 10.3390/bioengineering13050525

**Published:** 2026-04-30

**Authors:** Kahina Amara, Oussama Kerdjidj, Mohamed Amine Guerroudji, Shadi Atalla, Naeem Ramzan

**Affiliations:** 1 Centre for Development of Advanced Technologies, Algiers 16081, Algeria; kahina.amara88@gmail.com (K.A.); okerdjidj@cdta.dz (O.K.); mguerroudji@cdta.dz (M.A.G.); 2 College of Engineering and Information Technology, University of Dubai, Dubai P.O. Box 14143, United Arab Emirates; satalla@ud.ac.ae; 3 School of Computing, Engineering and Physical Sciences, University of the West of Scotland, Paisley PA1 2B, Scotland, UK

**Keywords:** arrhythmia, classification, imbalanced dataset, deep learning, balancing techniques, 3D visualisation

## Abstract

Cardiovascular diseases are among the most prevalent global health conditions, making the accurate diagnosis and classification of cardiac abnormalities crucial for effective treatment and patient management. While the electrocardiogram (ECG) is the primary tool for assessing cardiac electrical activity, its manual analysis is often time-consuming and susceptible to interpretive error. To address these limitations, this work proposes a comprehensive deep learning pipeline for the automated classification of arrhythmias, incorporating specific strategies to mitigate the challenge of imbalanced datasets. Furthermore, we introduce a novel three-dimensional (3D) visualisation framework that provides interactive, anatomically precise renderings of the heart regions implicated by the ECG classification, thereby delivering enhanced diagnostic insight. Our evaluation demonstrates that the proposed data balancing techniques yield significant performance gains, and under our current experimental setup, the results are competitive with or exceed several previously reported methods. We acknowledge that a more rigorous inter-patient cross-validation is needed to fully establish generalisation. The resulting 3D visualisations not only enable precise anatomical localisation of arrhythmia substrates but also serve as a powerful interactive tool for clinical practice and medical education.

## 1. Introduction

Cardiovascular diseases (CVDs) are the leading cause of death globally. In 2022 alone, they were responsible for an estimated 19.8 million deaths, representing approximately 32% of all global mortality. Of these CVD-related deaths, 85% were attributable to heart attacks and strokes [1]. The electrocardiogram (ECG) is a primary, non-invasive tool for diagnosing these conditions. ECG is a graphic representation of the heart’s electrical activity. This activity, generated by the heart’s specialised pacemaker and muscle cells, is recorded non-invasively using electrodes placed on the skin. Manually analysing ECG signals to characterise and classify heartbeats is a complex, time-consuming process that is highly dependent on clinical expertise. This subjectivity can lead to diagnostic errors and inconsistent interpretations. Furthermore, standard ECG devices have limited sensitivity for intermittent arrhythmias, as they can only capture irregularities that occur during the brief recording window. Additionally, the technique’s reliance on physical electrodes introduces challenges; improper placement can compromise data accuracy, while prolonged use often causes patient discomfort. These challenges have prompted significant research into automated diagnosis systems. Such systems can assist doctors by optimising their time and effort, allowing for more efficient and accurate interpretation of patient results. By streamlining the initial analysis, healthcare professionals can focus on critical decision-making and patient care. By learning hierarchical data representations through multi-layered networks, deep learning delivers a paradigm shift in ECG analysis and interpretation [2]. It powers systems capable of continuous, real-time monitoring and superior interpretation accuracy, which is critical for capturing scarcity arrhythmias. Moreover, Machine Learning and Deep Learning models introduce a much-needed standardisation to the diagnostic process, overcoming human variability to drive better patient outcomes. Fully realising this potential makes it imperative to endorse further research in the field. Qureshi et al. [3] proposed a deep learning approach utilising Convolutional Neural Networks (CNNs) for multi-class heartbeat categorisation. To enhance the model’s robustness, the authors incorporated regularisation techniques such as dropout and early stopping. They also addressed the issue of class imbalance through the Synthetic Minority Over-sampling Technique (SMOTE). This framework achieved an accuracy of 96% in classifying ten distinct arrhythmia categories from the MIT-BIH dataset [4].

In a separate study, Ahmed et al. [5] developed a 1D-CNN model for cardiac arrhythmia classification. A key aspect of their methodology was the use of data augmentation, where noise was intentionally added to the MIT-BIH signals during training to improve model generalisation. Using Lead II heartbeat data, their model classified heartbeats into four classes, achieving a notable accuracy of 99%. The model also demonstrated high performance in terms of recall (94%) and specificity (99%).

Mahmud et al. [6] introduced a hybrid approach that combined a 1D CNN for processing raw ECG signals with a 2D CNN, utilising transfer learning, for analysing ECG images. The predictions from both models were fused, yielding a robust classification system. Their method achieved 94% accuracy on ECG signals and 93% accuracy on images across five classes. The authors noted that results on the image data were further improved by augmenting the dataset.

Karri et al. [7] leveraged a Long Short-Term Memory (LSTM) network, a type of recurrent neural network (RNN) adept at modelling sequential data, for ECG classification. Using the MIT-BIH arrhythmia database, they developed a QRS complex detection technique based on discrete wavelet transform and delta-sigma modulation. The extracted features were then combined and fed into the LSTM. This approach achieved a high accuracy of 99.64% and an F1-score of 98.18% across five classes.

In a related study, Yildirim et al. [8] also employed an LSTM for classification but focused on a compressed feature space. They first used a convolutional autoencoder to create a compact representation of the ECG signals, which significantly reduced computational cost. The LSTM network subsequently classified the encoded features. This method demonstrated exceptional performance, achieving approximately 99% for both accuracy and F1-score on five classes of the MIT-BIH dataset.

Beyond medical imaging, CNNs have been successfully deployed in diverse real-world settings. In environmental monitoring, CNN-based segmentation has become the most practical approach for extracting real-time information from satellite and drone imagery, for example, in flash flood forecasting [9]. In industrial automation, lightweight 1D-CNN models running on ultra-low-power devices achieve near 98% accuracy for anomaly detection in manufacturing lines [10]. Furthermore, the growing need for real-time, privacy-preserving analysis has spurred the development of CNN accelerators for edge devices, such as FPGA-based implementations for wearable healthcare sensors [11], which aligns closely with the goals of the present work.

Recently, Kolmogorov–Arnold Networks (KANs) have emerged as a powerful alternative to traditional Multi-Layer Perceptrons (MLPs), offering superior accuracy and interpretability–two critical requirements in healthcare applications [12]. Unlike conventional fixed-activation networks, KANs replace weight parameters with learnable univariate spline functions, enabling them to approximate complex nonlinear relationships more effectively. In the context of physical systems, Peng et al. [13] demonstrated the effectiveness of KANs for predicting pressure and flow rate in flexible electrohydrodynamic (EHD) pumps, achieving significant performance gains over traditional models such as MLPs and Random Forest. Beyond engineering applications, KANs have shown considerable promise in medical diagnostics. For instance, Cui et al. [14] proposed KANs for ECG feature extraction and arrhythmia classification on the MIT-BIH database, achieving higher accuracy with lower computational complexity compared with conventional methods. Similarly, Zhao et al. [15] introduced MAK-Net, a multi-scale attentive KAN with BiGRU, which attained state-of-the-art performance (0.9980 accuracy, 0.9888 F1-score) for imbalanced ECG classification by synergistically applying focal loss and SMOTE. KANs have also been successfully applied to cardiovascular risk assessment, with Al Bataineh et al. [16] using a KAN to predict carotid intima-media thickness (CIMT), a key indicator of asymptomatic atherosclerosis, achieving 93% accuracy and outperforming six conventional algorithms. Furthermore, Pendyala and Venkatachalam [12] systematically evaluated KANs across multiple healthcare datasets, demonstrating that KANs outperform conventional ANNs in predictive accuracy, robustness, and interpretability, while enabling the extraction of symbolic expressions for transparent clinical decision-making. Collectively, these recent studies highlight the versatility and growing importance of KANs in biomedical signal processing and clinical diagnostics.

Deep learning models, particularly CNNs and LSTMs, have proven highly effective in classifying ECG data. Their performance is further enhanced by complementary techniques like data augmentation and domain adaptation. Nevertheless, significant challenges persist, such as tackling class imbalance and ensuring model generalisability across diverse patient demographics. The performance of deep learning models in accurate ECG classification for arrhythmia diagnosis is critically limited by the scarcity of diverse, well-annotated data. This challenge is compounded by the pronounced class imbalance inherent in public ECG datasets, where certain arrhythmias are significantly under-represented. Consequently, the development and application of robust dataset preprocessing techniques are essential to mitigate this imbalance and facilitate the training of reliable models. Future research must address these issues to advance the robustness and clinical applicability of automated arrhythmia diagnosis.

Unlike traditional medical education, which lacks depth perception and detail for small anatomical structures, 3D modelling and visualisation provide a comprehensive and accurate understanding of complex human heart anatomy. This technology has direct applications in clinical training, computational simulation of arrhythmias, and medical device development [17,18]. 3D heart modelling is revolutionising cardiovascular medicine by enabling precise anatomical visualisation. These technologies facilitate more accurate diagnoses and improve surgical planning. For example, patient-specific 3D-printed heart models can accurately replicate healthy and diseased cardiac structures. These models serve as invaluable tools, allowing surgeons to rehearse complex procedures and enabling the pre-operative customisation of medical devices.

According to the UK NHS, revolutionary AI-driven 3D heart scans are transforming cardiac care by enhancing efficiency [19]. This technology minimises invasive procedures, accelerates diagnostic timelines, and optimises resource allocation, thereby supporting clinicians in delivering more personalised treatment recommendations. In the domain of medical education, recent studies have developed innovative tools to improve the understanding of cardiac electrophysiology. For instance, ref. [20] proposed a user-friendly arrhythmia system for educational purposes. Their process involves receiving ECG signals and visualising the corresponding heartbeat activity. Using a CNN approach to classify four types of arrhythmias, the system reportedly achieved 99.7% accuracy on the MIT-BIH dataset. The authors claim the tool provides an enhanced understanding of cardiac activity and a more engaging learning experience. Complementing this, ref. [21] reported that 3D heart anatomical modelling improves learning efficacy. Ref. [21] proposed an interactive augmented reality mobile application that integrates a detailed 3D anatomical heart model with corresponding clinical ECG presentations to deepen understanding of cardiac electrophysiology. Based on [22], reformulating the standard 2D ECG into a 3D geometric representation uncovers electrophysiological information not visible in conventional projections. By employing 2D and 3D geometric descriptors with a deep-learning classifier (a residual multilayer perceptron), the authors demonstrated that integrating torsion into a 3D ECG framework enhances the detection of acute ischemia. This advancement increases diagnostic specificity and improves early triage and clinical decision-making in acute cardiac care.

The work by [23] presented a system for the digitisation and AI-based interpretation of ECG signals using an augmented reality headset. Their process begins with a printed ECG image, which is digitised to extract signal values. These values are then classified into different cardiomyopathy categories by a pre-trained deep learning algorithm, reportedly achieving an accuracy of 96.5%. The diagnostic result is subsequently visualised within the AR headset for user interpretation.

While the works mentioned above primarily focus on the 3D modelling of the human heart and ECG signal visualisation and its benefits for learning physiology, our research extends this field significantly, we propose a system that generates a 3D visualisation of the specific heart sub-regions responsible for arrhythmias, as identified by our deep learning-based ECG classification framework, which incorporates data-balancing approaches. This targeted visualisation provides a direct link between algorithmic diagnosis and anatomical reality, offering a powerful tool for both arrhythmia diagnosis aid and medical education and training.

The rest of the paper is organised as follows: Section 2 details our proposed methodology for arrhythmia classification. This includes a description of the dataset, the preprocessing techniques applied, the deep learning architectures employed, and the evaluation metrics used to assess performance. Subsequently, Section 2 presents our framework for 3D heart visualisation, showcasing the visual results and interactive features that facilitate the analysis of cardiac anomalies. Finally, the paper concludes in Section 3 with a summary of the key findings, a discussion of the study’s limitations, and an outline of potential future work to enhance clinical utility.

## 2. Proposal

**Figure 1:**
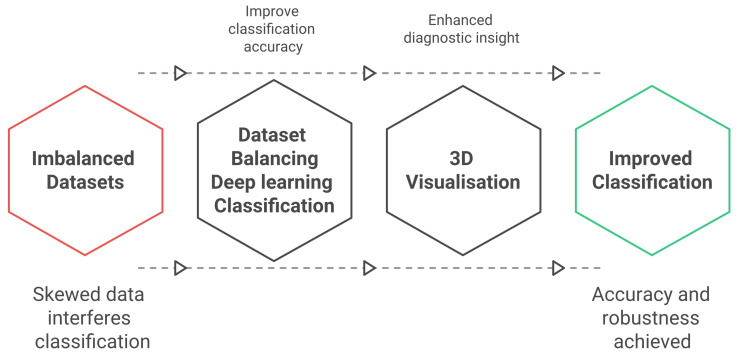
Proposal flowchart for Arrhythmia classification and 3D visualisation.

This section introduces an integrated framework for ECG classification, designed to handle class imbalance, 3D visualisation and details its component (Figure 1).

### 2.1. Arrhythmia Classification

#### 2.1.1. Used Dataset, Used Balanced Techniques

This section presents the dataset used for our study, detailing its properties and justifying its selection. For our research, we utilised the MIT-BIH Arrhythmia database [4], a benchmark dataset for developing and evaluating automated arrhythmia detection systems. Since its creation in 1980, it has served as a fundamental resource for cardiac rhythm research.

The dataset contains a sufficient volume of ECG samples to effectively train deep neural networks. These signals represent a wide range of normal heartbeats, arrhythmias, and myocardial infarctions. Each recording has been preprocessed and segmented into individual heartbeats, making it suitable for heartbeat classification tasks.

The primary challenge in automated ECG analysis lies in the signal’s complexity, including the diversity of rhythm waveforms and the presence of noise and artifacts. The MIT-BIH database encapsulates these challenges, providing a robust benchmarks for developing robust deep learning models. Our work leverages this dataset to explore deep neural network architectures and investigate the capabilities of balancing techniques in overcoming class imbalance and improving classification accuracy.

**Figure 2:**
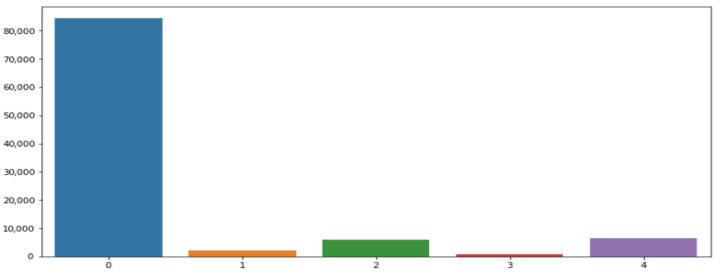
Distribution of classes in the MIT-BIH database.

The dataset comprises 109,446 samples, each described by 188 attributes. The first 187 columns (0 to 186) represent the input samples, while the final column (187) corresponds to the target label for classification. This label includes five categories (0 to 4) corresponding to different heartbeat types. An initial analysis confirmed the absence of missing or redundant values. However, the dataset exhibits a severe class imbalance, which is a critical consideration for model training. The distribution of classes is as follows (see Figure 2):

- Class 0 (Normal Rhythm): 84,363 samples
- Class 1 (Premature Supraventricular Beat): 2223 samples
- Class 2 (Premature Ventricular Contraction): 5788 samples
- Class 3 (Fusion of Ventricular and Normal Beat): 641 samples
- Class 4 (Unclassifiable Rhythm): 6431 samples

**Figure 3:**
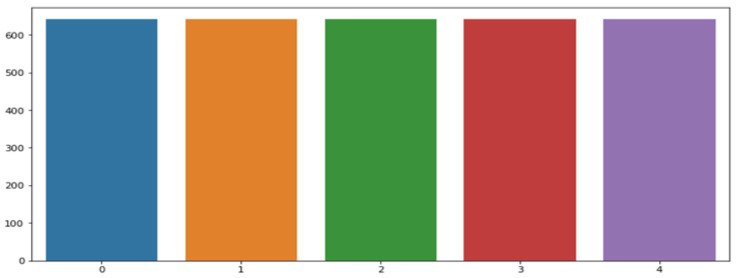
Distribution of training data after data downsampling.

To address class imbalance, we adopted two primary techniques: downsampling the majority classes and oversampling the minority classes. First, we performed random undersampling, reducing the size of all majority classes to match the minority class (’Fusion of Ventricular and Normal Beat,’ N = 641) (see Figure 3). The dataset was partitioned using a patient-wise split strategy. Training was conducted on 80% of the distinct patient records, while the remaining 20% of patients were reserved exclusively for final testing to prevent inter-patient data leakage and to assess true generalisation to unseen subjects. To mitigate class imbalance, we applied the Synthetic Minority Over-sampling Technique (SMOTE). This method creates synthetic examples rather than duplicating existing ones, which has been shown to improve model generalisation and reduce overfitting.

**Figure 4:**
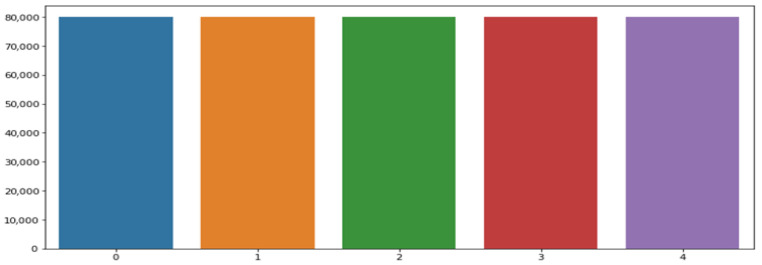
Distribution of training data after data oversampling.

Subsequently, an oversampling technique was applied to augment the minority classes, increasing their size to match that of the majority ’Normal Beat’ class (N > 80,000), as shown in Figure 4. Prior to feeding the data to the neural networks, each of the 187 input samples was normalised using Min–Max scaling to the range [0, 1]. The scaling parameters (minimum and maximum values) were computed exclusively from the training set and then applied to the validation and test sets.

#### 2.1.2. Used Models and Features Extraction

For the classification task, we use two DL models: Convolutional Neural Networks (CNNs) and A Multilayer Perceptron (MLP). The used CNN [24] consists of three 1D convolutional blocks, each followed by batch normalisation and max-pooling. The first convolutional layer uses 32 filters of size 5, producing an output shape of (183, 32) and containing 192 trainable parameters. After batch normalisation, a max-pooling layer with a pool size of 2 reduces the spatial dimension to 91. The second convolutional layer employs 64 filters of size 5, generating 10,304 parameters and an output shape of (87, 64), again followed by batch normalisation and max-pooling (output shape 43, 64). The third convolutional layer applies 128 filters of size 3, resulting in 24,704 parameters and an output shape of (41, 128); subsequent batch normalisation and max-pooling further compress the feature maps to (20, 128). The feature maps are then flattened into a vector of 2560 elements and passed to a dense layer with 128 units (327,808 parameters), a dropout layer (rate not specified in the table but typically 0.5 in similar architectures), and finally a dense output layer with 8 units (1032 parameters)—likely corresponding to the number of arrhythmia classes or diagnostic categories. This architecture balances model capacity with computational efficiency, using progressively more filters (32, 64, and 128) to capture higher-level abstractions while pooling reduces the temporal resolution and controls overfitting.

A Multi-Layer Perceptron (MLP) is a type of feedforward neural network that includes at least one hidden layer between its input and output layers [25]. It is intended for static (non-sequential) data, processing information in a single forward direction (input, hidden, and output). The input layer consists of as many neurons as there are features in the dataset. Hidden layers—usually one to three fully connected layers—typically contain 32 to 1024 neurons each, depending on the complexity of the data. The output layer has neurons equal to the number of target classes (10 for digit classification) or a single neuron for regression tasks. Nonlinear activation functions such as ReLU, Sigmoid, and Tanh are applied in the hidden layers; for classification, a Softmax activation converts logits into class probabilities, while regression tasks use a linear or sigmoid output. Several hyperparameters influence network performance, including the number of hidden layers (depth), the number of neurons per layer (width), and optimisation settings like learning rate (0.001) and the choice of optimiser (Adam, SGD).

The foundational step in our feature extraction pipeline was the accurate detection and parameterisation of the QRS complex, which represents the electrical signature of ventricular depolarisation and information-dense segment of the cardiac cycle. By transforming the raw ECG signal into these physiologically meaningful QRS-based descriptors, we provided the model with a robust and compact feature set that directly captures aberrations in cardiac electrophysiology associated with different arrhythmias, thus forming a critical basis for effective classification.

#### 2.1.3. Evaluation Metrics, Training, and Validation

The model is compiled with the following configuration: the *categorical crossentropy* loss function is used for this multi-class classification task. We selected the Adam optimiser due to its adaptive learning rate capabilities and proven efficiency. Model performance is evaluated using the accuracy metric. Prior to model training, we performed hyperparameter optimisation using a Grid Search to identify the most effective configuration for each algorithm. The search yielded the following optimal parameters: Batch Size: 40, Number of Epochs: 100.

Architectural hyperparameters (number of convolutional layers, number of filters per layer, kernel sizes, pooling sizes, dropout rate, and the inclusion of batch normalisation) were not optimised via grid search; they were determined based on standard practices in 1D-CNN designs for ECG classification.

Because this is a 5-class problem, binary metrics (Accuracy, MCC) were computed using a one-vs-rest strategy. The final reported values are macro-averaged over the five classes.

For model performance assessment, we used the following metrics: Accuracy (ACC) (Equation (1)), MCC (Matthews Correlation Coefficient) (Equation (2)), Mean Squared Error (MSE) (Equation (3)) with parameter definitions:

(1) $$\mathrm{ACC}=\frac{TP+TN}{TP+TN+FP+FN}$$

(2) $$MCC=\frac{TP\cdot TN-FP\cdot FN}{\sqrt{\left(TP+FP)(TP+FN)(TN+FP)(TN+FN\right)}}$$

where:

 TP=True Positives TN=True Negatives FP=False Positives FN=False Negatives

(3) $$MSE=\frac{1}{n}\sum_{i=1}^{n}{\left(y_{i}-{\hat{y}}_{i}\right)}^{2}$$

where:

 n=Number of data points yi=Actual value for the i-th data point y^i=Predicted value for the i-th data point

#### 2.1.4. Classification Performance

The models were initially trained on the raw, imbalanced dataset without applying any data balancing techniques. The CNN model achieved superior results across all evaluated metrics. It attained a classification accuracy (ACC) of 98.56%, marginally higher than the MLP’s 97.98%. While this difference may appear small, it is substantiated by the more significant disparity in the Mean Squared Error. The CNN’s markedly lower MSE (0.0556 versus 0.11 for the MLP) indicates a more precise and confident prediction model with reduced average prediction error. Most conclusively, the Matthews Correlation Coefficient (MCC), which provides a balanced measure especially important for imbalanced datasets, was highest for the CNN at 94.20%, compared with 92.28% for the MLP. The MCC consolidates true and false positives and negatives into a single metric, and its higher value for the CNN confirms a more robust overall classification performance beyond simple accuracy. The downsampling pretreatment enabled the MLP model to achieve an accuracy of 89.09%, a mean squared error (MSE) of 0.32, and a Matthews correlation coefficient (MCC) of 85.62%. The CNN model trained on downsampled data achieved an accuracy of 98.52%, a mean squared error (MSE) of 0.0635, and a Matthews correlation coefficient (MCC) of 94.12%.

The MLP model achieved exceptional performance on the oversampled dataset, with an accuracy of 99.07%, mean squared error (MSE) of 0.0456, and Matthews correlation coefficient (MCC) of 98.66%. Similarly, the CNN model trained on oversampled data demonstrated strong results, yielding 98% accuracy, 0.06 MSE, and 93% MCC.

The comparative results presented in Table 1 provide insights into the interplay between model architecture and data balance for ECG arrhythmia classification. The performance, evaluated through Accuracy (ACC), Mean Squared Error (MSE), and Matthews Correlation Coefficient (MCC), reveals distinct trends that underscore the importance of selecting both the right model and the appropriate data handling technique.

On the original, imbalanced dataset, a clear hierarchy of model capability emerges. The CNN achieves the highest performance (ACC: 98.56%, MCC: 94.20%), closely followed by the MLP (ACC: 97.89%, MCC: 92.28%). This superior performance can be attributed to their inherent strengths: the CNN’s ability to extract local, translation-invariant features from the ECG signal, and the MLP’s capacity to learn complex nonlinear mappings from the 187 input samples.

The application of undersampling, a technique to combat class imbalance, yields divergent outcomes. For the MLP, performance drops substantially (ACC: 89.09%, MCC: 85.62%), with MSE increasing over fivefold. This confirms the expected drawback of undersampling: the loss of a significant amount of data from the majority class critically impairs the model’s ability to learn generalisable patterns. Conversely, the CNN demonstrates remarkable robustness, maintaining near-identical accuracy and MCC (98.52%, 94.12%) compared with its performance on the initial dataset. This suggests that the CNN’s feature extraction is efficient enough that it can achieve high performance even with a severely reduced dataset, likely by focusing on the most discriminative local patterns present in all classes.

The most striking result emerges from the oversampled data. Here, the MLP becomes the top-performing model, achieving the highest scores in all reported metrics (ACC: 99.07%, MCC: 98.66%, and the lowest MSE: 0.0456). The MCC, which is a robust metric for imbalanced class scenarios, shows a dramatic improvement to 98.66%, indicating an excellent balance between sensitivity and specificity across all classes. This demonstrates that providing a balanced dataset through oversampling allows the simpler MLP architecture to learn optimal decision boundaries without being biased by the initial class distribution. The CNN’s performance with oversampling remains excellent but does not show a similar improvement (ACC: 98.54%, MCC: 93.96%), potentially due to a minor effect of overfitting on the synthetic data.

The evaluation study of the four deep learning models reveals critical insights into the interplay between model architecture and data balance for ECG classification. The analysis leads to two primary conclusions for this specific task. First, the CNN is the most robust model, delivering state-of-the-art performance regardless of significant data reduction through undersampling. Second, when paired with an effective data balancing technique like oversampling, the MLP can achieve the absolute best performance, surpassing even the robust CNN.

The performance of the proposed models is benchmarked against recent state-of-the-art deep learning approaches for ECG classification in Table 2. The results demonstrate that our methodology is highly competitive, with one configuration establishing a new performance benchmark. Our proposed MLP model with oversampling achieves the highest accuracy among all compared methods at 99.07%. This surpasses the previous high of 98.72% reported by [26] for an MLP architecture, and outperforms advanced CNN variants such as the RD-CNN from [27] (98.5%) and the CNN from [28] (98.45%). This indicates that a well-regularised MLP, when trained on a balanced dataset, can outperform more complex convolutional architectures for this specific task, likely due to its ability to learn optimal decision boundaries across all classes without being biased by the initial class distribution. Furthermore, our standard CNN model, without any specialised architectural modifications, achieves a robust accuracy of 98.56%. This performance is competitive with, and even marginally exceeds, the dedicated CNN models presented in [29] (97%) and [28] (98.45%). This confirms the inherent strength and generalisability of the convolutional approach for feature extraction from ECG signals, validating our architectural choices. In conclusion, while our CNN implementation delivers state-of-the-art performance, the superior result from the MLP with oversampling underscores a critical finding: for ECG arrhythmia classification, the strategic handling of class imbalance can be as impactful as the choice of the model architecture itself. Our best-performing model not only validates the efficacy of our preprocessing pipeline but also sets a new benchmark for accuracy in this domain.

### 2.2. 3D Visualisation for Arrhythmia Aid Diagnosis

Although deep learning models have advanced automated classification, their “black-box” nature can obscure the underlying pathophysiology, creating a disconnect between algorithmic output and clinical understanding. To bridge this gap, we develop a novel 3D visualisation platform designed to transform arrhythmia diagnosis from signal analysis into an immersive, intuitive, and spatially-aware experience. This system translates the output of our high-accuracy deep learning classifier into a dynamic, interactive 3D visualisation of the human heart, along with intuitive interaction. Clinicians can employ an AR headset to inspect a 3D virtual heart model in their physical environment, leveraging voice commands and hand-tactile interactions to naturally zoom, rotate, and explore its anatomy. Crucially, upon identifying an arrhythmia, the system automatically highlights and annotates the specific heart sub-regions, such as the sinoatrial node, atria, or ventricles, responsible for the detected anomaly. This direct mapping from ECG classification to a 3D visual representation of the malfunctioning cardiac substrate provides an unparalleled pedagogical and diagnostic tool, fostering a deeper understanding of the arrhythmia’s origin and mechanism, and ultimately paving the way for more precise and informed clinical decision-making.

#### 2.2.1. 3D Visualisation, Interaction

**Figure 5:**
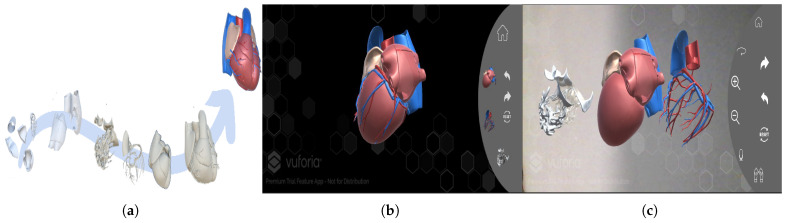
3D Heart visualisation: (**a**) Assembly of 152 Anatomical Components in a 3D Modelling Environment, (**b**) A Digital Reconstruction and visualisation of the Human Heart, (**c**) 3D Heart sub-regions decomposition.

The construction of detailed 3D anatomical models from digital parts has gained significant traction across multiple disciplines. For our system, the foundational 3D heart model was built using the BodyParts3D database, a comprehensive resource providing 152 distinct anatomical components of the human heart [30]. The primary challenge involved the precise spatial assembly of these interlocking parts into a coherent 3D structure. This required meticulous consideration of each component’s unique geometry and its correct position and orientation in 3D space. To elucidate this intricate process, we have included visual illustrations of the constituent parts from the BodyParts3D database and sequential images of the assembly, highlighting the precision and complexity inherent in creating a high-fidelity anatomical model (Figure 5).

The platform features an interactive 3D visualisation interface that enables immersive exploration of cardiac anatomy. Users can intuitively manipulate the model by zooming to focus on specific structures, rotating it through a full 360 degrees for multi-angle inspection, and decomposing it into constituent sub-models to isolate and study major anatomical regions.

**Figure 6:**
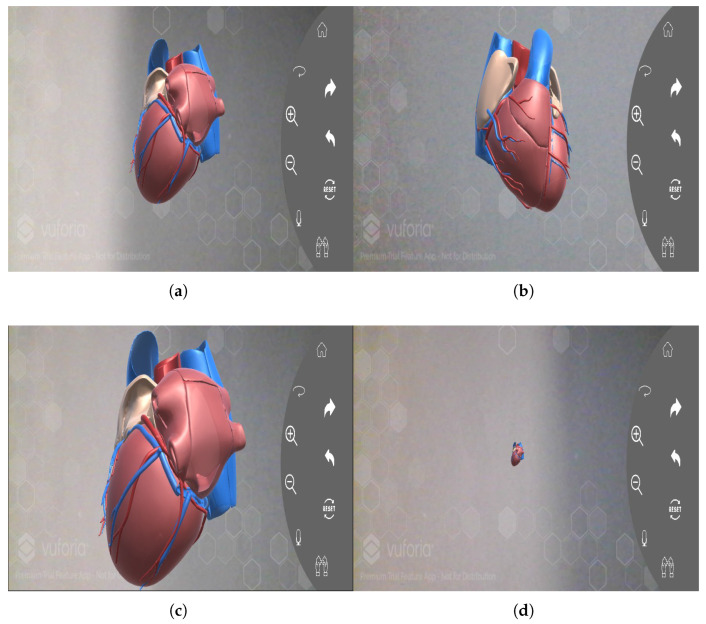
Interactive 3D visualisation: (**a**) 3D heart view, (**b**) Rotation, (**c**) Zoom-in, (**d**) Zoom-out.

Figure 6 illustrates the core interactive functionalities of the 3D heart visualisation system, which facilitates an immersive exploration of cardiac anatomy and arrhythmia manifestations. Figure 6a presents the default 3D heart view, offering an initial holistic representation. Figure 6b demonstrates the rotation feature, allowing the model to be examined from any angle to build a comprehensive spatial understanding. Figure 6c,d highlight the dynamic zoom capability, enabling users to inspect fine anatomical details of specific regions and then zoom out to re-contextualise these details within the entire organ. Collectively, these features transform static data into a dynamic diagnostic tool, enabling intuitive analysis of the heart’s complex geometry and the underlying substrates of cardiac arrhythmias.

#### 2.2.2. ECG Classification Visualisation Results: 3D Rendering

**Figure 7:**
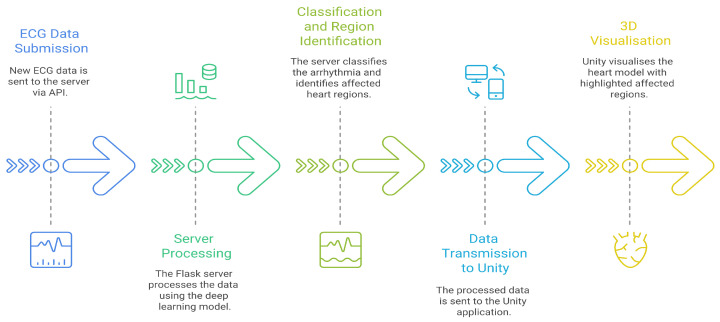
3D-Enhanced ECG Analysis Process.

The deployment of our deep learning ECG classification model into a 3D engine environment is facilitated through a client-server architecture. A robust Flask server [31], acts as the central processing unit, hosting the trained deep learning model. When new ECG data is submitted via a RESTful API, the server performs inference. It returns not just the arrhythmia classification, but also the specific heart sub-regions implicated in the anomaly. A Unity 3D application seamlessly consumes this data [32], which serves as the AR rendering engine. Within Unity, the diagnostic results dynamically trigger the visualisation of an anatomically precise 3D heart model. The affected sub-regions, such as the sinoatrial node or ventricular myocardium, are visually highlighted in the user’s physical space, allowing clinicians to intuitively understand the pathophysiology behind the arrhythmia through an immersive, interactive 3D experience that bridges the gap between abstract data and tangible anatomy (Figure 7).

**Figure 8:**
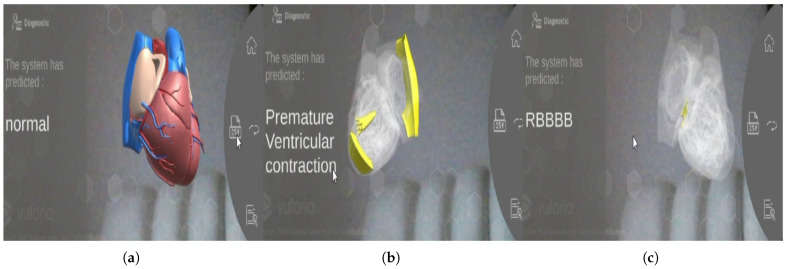
3D Visualisation of ECG Classification Results: (**a**) Normal Sinus Rhythm, (**b**) Premature Ventricular Contraction, (**c**) RBBB Right Bundle Branch Block.

Figure 8 provides a direct visual mapping of the deep learning model’s ECG classification output onto an anatomical 3D heart model. This visualisation paradigm transforms abstract arrhythmia diagnoses into an intuitive, spatially-accurate representation, aiding in the interpretation of results by highlighting the affected cardiac structures. The figure presents three distinct cases: Figure 8a a Normal Sinus Rhythm, where the heart model is displayed in its baseline state without anomalies; Figure 8b a Premature Ventricular Contraction (PVC), where the visualisation pinpoints the ectopic focus within the ventricular myocardium, explaining the origin of the premature beat; and Figure 8c a Right Bundle Branch Block (RBBB), which illustrates the conduction delay by emphasising the right ventricle and the corresponding bundle branch. By correlating the classified arrhythmia with its anatomical substrate, this 3D visualisation bridges the gap between classification output and clinical understanding, offering a powerful tool for education and diagnostic assistance.

## 3. Conclusions, Future Works

In conclusion, this research addresses critical challenges in cardiac care by developing an integrated system that combines robust automated diagnosis with intuitive anatomical visualisation. We have presented a comprehensive deep learning pipeline for arrhythmia classification that explicitly tackles the pervasive issue of imbalanced datasets, demonstrating superior accuracy and robustness compared with existing state-of-the-art methods. Beyond classification, our work introduces a novel 3D visualisation framework that bridges the gap between algorithmic output and clinical understanding. By generating interactive patient-specific renderings of the heart regions implicated in arrhythmia, this tool provides tangible anatomical context, improving diagnostic insight, and facilitating precise location of cardiac abnormalities. Together, these contributions form a synergistic approach that advances the field of automated cardiac analysis. The proposed system has significant potential as a dual-purpose platform: as a decision-support tool to improve diagnostic efficiency and accuracy in clinical practice, and as an immersive educational tool to train medical professionals in cardiac electrophysiology.

Several limitations should be acknowledged. First, our current model was developed and validated using only single-lead ECG data from the MIT-BIH database. Its generalisability to multi-lead ECG recordings (12-lead) remains to be established, as multi-lead signals may provide additional spatial information that could alter arrhythmia classification patterns. Second, although the dataset includes common arrhythmia types, rare arrhythmias (Brugada syndrome, long QT syndrome) are underrepresented. The performance of the model on such rare events has not been evaluated. Future work will focus on validating the framework with larger, multi-centre clinical datasets and exploring its real-time integration into clinical workflows to assess the impact on patient management and outcomes. Furthermore, we aim to conduct a formal evaluation study that incorporates feedback from cardiologists and clinical practitioners to assess the usability and diagnostic utility of the system. Beyond clinical validation, several technical and translational avenues will be pursued. First, we plan to extend the framework to multi-modal data fusion, combining ECG signals with other physiological measurements (photoplethysmography, blood pressure) and structured electronic health records (age, comorbidities) to improve risk stratification. Second, we will optimise the CNN architecture for edge deployment, targeting ultra-low-power microcontrollers and FPGA-based accelerators, enabling real-time arrhythmia detection in wearable devices with minimal latency and energy consumption. Third, we will conduct a dedicated end-user evaluation involving cardiologists, emergency physicians, and medical trainees to assess the system’s usability, diagnostic confidence, and integration into real-world clinical decision-making. Finally, leveraging longitudinal patient data, we will investigate personalised arrhythmia risk profiling to adapt the model to individual patient dynamics, potentially enabling early warning systems for acute cardiac events. These future directions aim to transition the proposed system from a proof-of-concept into a clinically deployable, patient-centred tool that enhances both diagnostic accuracy and workflow efficiency.

## Figures and Tables

**Table 1:** Macro-averaged Classification results of Deep Learning Models on the Original, Undersampled, and Oversampled ECG Datasets.

Dataset	Model	ACC (%)	MSE	MCC (%)
**Initial dataset**	CNN	98.56	0.0556	94.20
	MLP	97.89	0.11	92.28
**Dataset with Downsampling**	CNN	98.52	0.0653	94.12
	MLP	89.09	0.3212	85.62
**Dataset with Oversampling**	CNN	98.54	0.0524	93.96
	MLP	99.07	0.0456	98.66

**Table 2:** Performance Comparison of the Proposed Model with State-of-the-Art Deep Learning Methods.

Methods	Model	Performance
[29]	CNN	ACC. 97%
[28]	CNN	ACC. 98.45%
[26]	MLP	ACC. 98.72%
[27]	RD-CNN	ACC. 98.5%
**Our Proposal**	**MLP**	**ACC. 99.07%**
	**CNN**	**ACC. 98.56%**

## Data Availability

The dataset used in this study is publicly available at: https://www.kaggle.com/datasets/mondejar/mitbih-database (accessed on 21 April 2026).
